# High-affinity CD16 integration into a CRISPR/Cas9-edited CD38 locus augments CD38-directed antitumor activity of primary human natural killer cells

**DOI:** 10.1136/jitc-2021-003804

**Published:** 2022-02-08

**Authors:** Joseph Andrew Clara, Emily R Levy, Robert Reger, Stefan Barisic, Long Chen, Elena Cherkasova, Mala Chakraborty, David S J Allan, Richard Childs

**Affiliations:** 1 Laboratory of Transplantation Immunotherapy, Cellular and Molecular Therapeutics Branch, National Heart Lung and Blood Institute, National Institutes of Health, Bethesda, Maryland, USA; 2 Biologics Process Research and Development, Merck & Co Inc, Kenilworth, New Jersey, USA

**Keywords:** immunotherapy, killer cells, natural, cell engineering, cytotoxicity, immunologic

## Abstract

**Background:**

Adoptive transfer of natural killer (NK) cells with augmented antibody-dependent cellular cytotoxicity (ADCC) capabilities and resistance to CD38 targeting has the potential to enhance the clinical anti-myeloma activity of daratumumab (DARA). Therefore, we sought to develop an efficient CRISPR/Cas9-based gene editing platform to disrupt CD38 expression (CD38 knockout (KO)) in ex vivo expanded NK cells and simultaneously arm CD38^KO^ NK cells with a high-affinity CD16 (CD16-158V) receptor.

**Methods:**

CD38^KO^ human NK cells were generated using Cas9 ribonucleoprotein complexes. The platform was expanded by incorporating messenger RNA (mRNA) transfection of CD38^KO^ NK cells and targeted gene insertion at the *CD38* locus to mediate gene knockin (KI). The capacity of these gene-edited NK cells to persist and mediate ADCC in the presence of DARA was tested in vitro and in a MM.1S xenograft mouse model.

**Results:**

Highly efficient CD38 gene disruption was achieved in ex vivo expanded NK cells without affecting their proliferative or functional capacity. CD38 KO conferred resistance to DARA-induced NK cell fratricide, enabling persistence and augmented ADCC against myeloma cell lines in the presence of DARA in vitro and in a MM.1S xenograft mouse model. CD38^KO^ NK cells could be further modified by transfection with mRNA encoding a CD16-158V receptor, resulting in augmented DARA-mediated ADCC. Finally, we observed that a homology-directed repair template targeted to the *CD38* locus facilitated an efficient 2-in-1 CD38 KO coupled with KI of a truncated CD34 reporter and CD16-158V receptor, with CD38^KO^/CD16^KI^ NK cells demonstrating a further enhancement of DARA-mediated ADCC both in vitro and in vivo.

**Conclusions:**

Adoptive immunotherapy using ex vivo expanded CD38^KO^/CD16^KI^ NK cells has the potential to boost the clinical efficacy of DARA. By incorporating complementary genetic engineering strategies into a CD38 KO manufacturing platform, we generated NK cells with substantially augmented CD38-directed antitumor activity, establishing a strong rationale for exploring this immunotherapy strategy in the clinic.

## Introduction

Natural killer (NK) cells are innate lymphocytes that play an important role in the immune defense against cancer. They have the unique capacity to recognize and kill tumor cells, in an antigen-independent manner, without prior sensitization.^1 2^ In recent years, efforts have focused on harnessing the innate antitumor properties of NK cells as a form of cancer immunotherapy. NK cells have been implicated in the activity of several anticancer therapeutic agents^3–6^ and have induced antitumor responses in the setting of adoptive NK cell transfer.^7–9^ The clinical exploration of NK cell-based therapy has been bolstered by the development of robust ex vivo expansion protocols^10^ that can generate large numbers of cytotoxic NK cells as well as a proven track record of safety.^7–9^ Advances in genetic engineering to enhance NK cell activity^11–14^ and novel combination strategies pairing NK cells with other types of anticancer agents^6 15 16^ and/or NK cell checkpoint inhibitors^17 18^ have resulted in NK cell-based therapeutics moving forward at a rapid pace. NK cells are primary effectors of antibody-dependent cellular cytotoxicity (ADCC), a key mechanism of action of several therapeutic monoclonal antibodies (mAbs) that function, in part, by directing NK cell cytotoxic activity to tumor targets.^19^ The potent NK cell activating receptor CD16 (FCGR3A) triggers ADCC on binding the constant region (Fc) of antibody-coated target cells.^19^ Combining adoptive NK cell infusions with mAb treatment represents one promising combination strategy that aims to augment the capacity of NK cells to mediate ADCC.^15 16^

Daratumumab (DARA) is a CD38-targeting human IgG1 mAb that has demonstrated clinical efficacy in the treatment of multiple myeloma (MM), both as a single agent and in combination regimens.^20 21^ DARA exerts its antitumor effects via multiple mechanisms of action, including potent NK cell-mediated ADCC,^5^ making DARA an attractive agent to combine with adoptive NK cell infusions. However, because peripheral blood NK cells have high levels of CD38 expression, DARA treatment may have a deleterious effect on NK cells. Several studies have shown that DARA leads to rapid depletion of NK cells in patients^22^ and in murine models by inducing CD16/ADCC-dependent NK cell fratricide.^23 24^ This effect may impair the NK cell-mediated ADCC activity of DARA and lead to a submaximal antitumor response. Preventing DARA from binding to CD38 on NK cells protects them from DARA-induced fratricide.^23 24^ Further, permanent disruption of CD38 expression (CD38 knockout (KO)) in primary NK cells via CRISPR/Cas9 editing has been shown to confer resistance to DARA-induced fratricide and augment DARA-directed ADCC.^25^

An adoptive NK cell strategy combining CD38^KO^ NK cells with DARA has the potential to enhance the antitumor responses of this mAb by enabling NK cell persistence and maximizing DARA’s ADCC effect. We and others have reported efficient gene deletion using non-viral Cas9 ribonucleoprotein (Cas9/RNP) complexes,^26^ and therefore, investigated the use of this technology to disrupt CD38 expression in primary ex vivo expanded NK cells from humans that were propagated in conditions mimicking those used for our phase I clinical trial (NCT00720785). We developed an efficient CRISPR gene editing platform to disrupt the expression of CD38, which reduced DARA-mediated NK cell fratricide, improved NK cell persistence in the presence of DARA, and augmented DARA-driven ADCC against CD38-expressing MM cell lines. To further augment the antitumor activity of CD38^KO^ NK cells with DARA, we transfected CD38^KO^ NK cells with messenger RNA (mRNA) encoding the high-affinity CD16 (CD16-158V) receptor to boost their ability to mediate ADCC. Lastly, we designed a strategy to combine gene knock-in (KI) with our efficient CD38 KO platform by codelivering a homology-directed repair (HDR) template targeted to the *CD38* locus. As proof of concept, we used this methodology for targeted insertion of a selectable reporter protein, truncated CD34. Our streamlined, 2-in-1 KO/KI method resulted in KO of CD38 in 92% of NK cells with concurrent CD34 KI in 85% of CD38^KO^ NK cells. We also utilized this strategy to enhance the functional capabilities of CD38^KO^ NK cells by inserting a FLAG-tagged CD16-158V receptor, which resulted in detectable FLAG expression in approximately 57% of CD38^KO^ NK cells that had increased DARA-mediated ADCC against MM tumor targets. In summary, we present effective gene editing strategies that have the potential to synergistically enhance the DARA-mediated antitumor activity of ex vivo expanded NK cells when combined with CRISPR/Cas9 deletion of CD38.

## Materials and methods

### Cell lines and reagents

The K562, Raji, NCI-H929, and MM.1S cell lines were purchased from American Type Culture Collection. The EJM myeloma cell line was provided to the Childs’ lab by Leslie Brents, Walter Reed National Military Medical Center (Bethesda, Maryland, USA). The Epstein–Barr virus-transformed lymphoblastoid cell line (EBV-LCL) SMI-EBV-LCL was previously described.^27^ Cell lines were propagated in RPMI 1640 medium (Gibco, Gaithersburg, Maryland, USA) supplemented with 10% heat-inactivated fetal bovine serum (Millipore-Sigma, Massachusetts, USA).

### Flow cytometry

NK cells were stained with antibodies recognizing CD56 (NCAM16.2), CD3 (UCHT1), CD16 (3G8), CD38 (HIT2), CD34 (8G12), NKp44 (P44-8.1), KIR3DL1 (HP-3E4), KIR2DL1 (HP-3E4), CD107a (H4A3), TNF-α (6401.1111), and IFN-γ (4S.B3) from BD Biosciences (Franklin Lakes, New Jersey, USA), DNAM-1 (11A8), 2B4 (2–69), NKG2D (1D11), NKp30 (p30-15), NKp46 (9E2), CD62L (DREG-56), CXCR4 (12G5), Lir-1 (GHI/75), TRAIL (RIK-2), and FLAG tag (L5) from BioLegend (San Diego, California, USA), NKG2A (Z199) from Beckman Coulter (Brea, California, USA), NKG2C (134591) from R&D Systems (Minneapolis, Minnesota, USA) along with annexin V (BioLegend), and/or a Live/Dead fixable aqua dead cell stain kit (Thermo Fisher Scientific, Waltham, Massachusetts, USA). Flow cytometry was performed using a LSRFortessa instrument (BD Biosciences), and data were analyzed with FlowJo V.10.1 software (BD Biosciences).

### Isolation and culture of primary human NK cells

Deidentified healthy donor peripheral blood buffy coats were provided by the Department of Transfusion Medicine, NIH Clinical Center. NK cells were isolated using RosetteSep human NK cell enrichment cocktail (STEMCELL Technologies, British Columbia, Canada) with lymphocyte separation medium (MP Biomedicals, Irvine, California, USA) or by CD3 depletion, followed by CD56 selection using magnetic-activated cell sorting beads (Miltenyi Biotec, Bergisch Gladbach, Germany). NK cells were combined with irradiated SMI-EBV-LCL cells at a ratio of 1:10 in NK cell media [X-VIVO 20 medium (Lonza, Basel, Switzerland) supplemented with 10% heat-inactivated human AB serum (Millipore-Sigma), 2 mM GlutaMAX (Thermo Fisher Scientific), and 500 IU/mL IL-2 (teceleukin, Roche, Basel, Switzerland, provided by NIH/NCI, Fredrick, Maryland, USA). The cells were cultured at 37°C and 6.5% CO^2^. Half of the media was replaced 5 days into the expansion and NK cells were subsequently counted and cell concentrations adjusted to 0.5–1×10^6^ cells/mL every 48 hours until utilized in experiments.

### Electroporation of NK cells

To generate CD38^KO^ NK cells we used a gRNA (AGUGUAUGGGAUGCUUUCAA) to target the second exon of the *CD38* gene, designed using the Synthego CRISPR design tool (https://design.synthego.com/) and purchased from Synthego (Redwood City, California, USA) as a modified gRNA. Cas9 nuclease (Alt-R S.p. HiFi Cas9 Nuclease V.3) was purchased from Integrated DNA Technologies (Coralville, Iowa, USA). Primary NK cells were expanded ex vivo using irradiated SMI-EBV-LCL feeder cells as described above. On day 7, NK cells were collected and washed in electroporation buffer (Hyclone, MaxCyte, Gaithersburg, Maryland, USA). They were then mixed with precomplexed Cas9/RNPs (1 µM Cas9, 5 µM gRNA) in a total volume of 100 uL and electroporated with the Maxcyte ATX Transfection System using the preprogrammed NK-04 eleprotocoporation protocol. NK cells were recovered in warm NK cell media. For KI experiments, viral particles containing recombinant adeno-associated virus pseudotyped with AAV6 serotype envelope (rAAV6) were added to NK cells 30 min following electroporation at a multiplicity of infection of 150 000. AAV particles were manufactured by VectorBuilder (Chicago, Illinois, USA) according to our design. For mRNA transfection, NK cells were collected on day 14 of expansion and the Maxcyte ATX Transfection System was used to deliver 4 µg mRNA/1×10^6^ NK cells as previously reported.^11^ Custom-made CD16-158V mRNA was obtained from TriLink Biotechnologies (San Diego, California, USA).

### PCR to confirm site-specific transgene integration

Genomic DNA was extracted from NK cells 7 days postelectroporation using DNeasy Blood and Tissue Kit (Qiagen, Germantown, Maryland, USA). Genomic DNA was then then subjected to PCR using HotStarTaq DNA polymerase (Qiagen) according to the manufacturer’s protocol with primers designed to span and amplify the 3’ junction of the inserted CD34 transgene and genomic CD38 (forward 5’-AACTTGATCGTGGTGGATAAGC-3’, reverse 5’-TTCCATTCCTACCAACCACACT-3') or the 3’ junction of inserted CD16-158V and genomic CD38 (forward 5’-ATTGGGAAGAGAATAGCAGGCA-3’, reverse 5’-CACGCCTAGTGAATGGAGAAGT-3’). Samples were then run on a 1% agarose gel and imaged using the Amersham Imager 680 (GE Healthcare, Chicago, Illinois, USA).

### Analysis of CD38-targeting guide RNA off-target activity

Based on a previously reported strategy,^28^ the human genome was queried for potential Cas9 off-target sites using the Cas-OFFinder search tool^29^ (http://www.rgenome.net/cas-offinder/) with the CD38-targeting gRNA from this study as a query sequence. Up to three mismatched base pairs between the gRNA and potential off-target sites were allowed. A total of fifteen potential off-target sites were identified that harbored three mismatches, of which five were randomly chosen for further analysis. No sites were detected with only one or two mismatches. The off-target sites and CD38-specific on-target site were PCR amplified as described above from bulk genomic DNA from CD38^WT^ and CD38^KO^ NK cells (primers are shown in online supplemental table S1). PCR products were purified using the QIAquick PCR Purification Kit (Qiagen) and Sanger sequenced (Eurofins Genomics, Luxembourg; primers are shown in online supplemental table S1). To determine the presence of insertions and/or deletions (indels) at the on-target and off-target sites, Sanger sequencing data was analyzed using the on-line inference of CRISPR Edits (ICE) tool (Synthego; available at https://ice.synthego.com).^30^

10.1136/jitc-2021-003804.supp1 Supplementary data

### NK cell functional assays

NK cells were cocultured with K562 target cells or Raji cells at a 1:1 ratio in the presence or absence of media containing 10 µg/mL of rituximab. To assess degranulation that occurred as a consequence of fratricide, NK cells were cultured alone with media containing 10 µg/mL of DARA. Degranulation was assessed at 4 hours by CD107a surface staining for flow cytometry. In parallel cultures using the same above conditions, cytokine production was examined at 6 hours. Brefeldin A and monensin (BD Biosciences) were added at recommended concentrations within the first hour and cells were incubated for an additional 5 hours. Cells were then fixed and permeabilized using Cytofix/Cytoperm reagents (BD Biosciences) before intracellular staining for cytokine production. Cell viability during fratricide experiments was determined by staining with annexin V (BioLegend) and a Live/Dead fixable aqua dead cell stain kit (Thermo Fisher Scientific).

### Target cell killing assays

NK cells were cocultured at various effector-to-target ratios with calcein AM-labeled MM.1S, NCI-H929, or EJM myeloma cells seeded at 10 000 target cells/well, in the presence or absence of media containing 10 µg/mL of DARA in a final volume of 200 µL in 96-well plates at 37°C and 5% CO^2^. After 4 hours, wells were mixed and 100 µL from each well was transferred to a black-walled flat bottom 96-well plate and centrifuged before counting on a Celigo Image Cytometer (Nexcelom, Lawrence, Massachusetts, USA). Specific target lysis was calculated using the following formula: [(1 − (experimental calcein AM positive/spontaneous calcein AM positive)) × 100]. Data given are the means of triplicate determinations.

### Persistence of adoptively transferred human NK cells in NSG mice

NOD-*scid* IL2Rgamma^null^ (NSG) mice aged 8–10 weeks were purchased from Jackson Laboratories (Bar Harbor, Maine, USA). Ex vivo expanded CD38^KO^ and CD38^WT^ NK cells were harvested on day 14 and injected via tail vein into NSG mice at a dose of 1×10^7^ cells/animal. Mice were administered DARA 10 µg or saline as a control intraperitoneally on the same day. Twenty-four hours later, mice were humanely euthanized and bone marrow, peripheral blood, liver, and spleen were harvested from each mouse and analyzed by flow cytometry for the presence of human NK cells.

### MM mouse model

MM.1S cells were lentivirally transduced to express firefly luciferase with a lentiviral vector encoding EGFP, T2A self-cleaving peptide, and humanized firefly luciferase, with GFP-positive cells subsequently purified by FACS. NSG mice were inoculated with 5×10^6^ luciferase-expressing MM.1S cells via tail vein injection on day 0, then on days 3, 10, and 17 were administered either DARA alone at a dose of 10 µg delivered via intraperitoneal (i.p.) injection, or 1×10^7^ ex vivo expanded CD38^WT^ or CD38^KO^ NK cells via tail vein injection along with 10 µg i.p. DARA. 50 000 IU of i.p. IL-2 was administered daily for 3 days starting on the day of each NK cell infusion. The identical treatment schedule and dosing regimen was conducted in a final experiment in mice comparing treatment with either CD38^KO^/CD16^KI^ or CD38^KO^ NK cells in combination with DARA. To monitor tumor burden, mice were injected with i.p. D-luciferin (150 mg/kg; Gold Biotechnology, St. Louis, Missouri, USA) and in vivo bioluminescence imaging was performed using In Vivo Imaging System (Spectrum) with Living Image software (PerkinElmer, Waltham, Massachusetts, USA).

### Statistical analysis

All data were analyzed with Prism V.9 software (GraphPad Software). Student’s t-test was used to test for significant differences between two independent groups. In vitro assays were repeated in three to eight independent donors. A p<0.05 was considered statistically significant. Mean values±SEM are shown.

## Results

### High-efficiency CD38 gene disruption does not compromise NK cell function or phenotype

**Figure 1:**
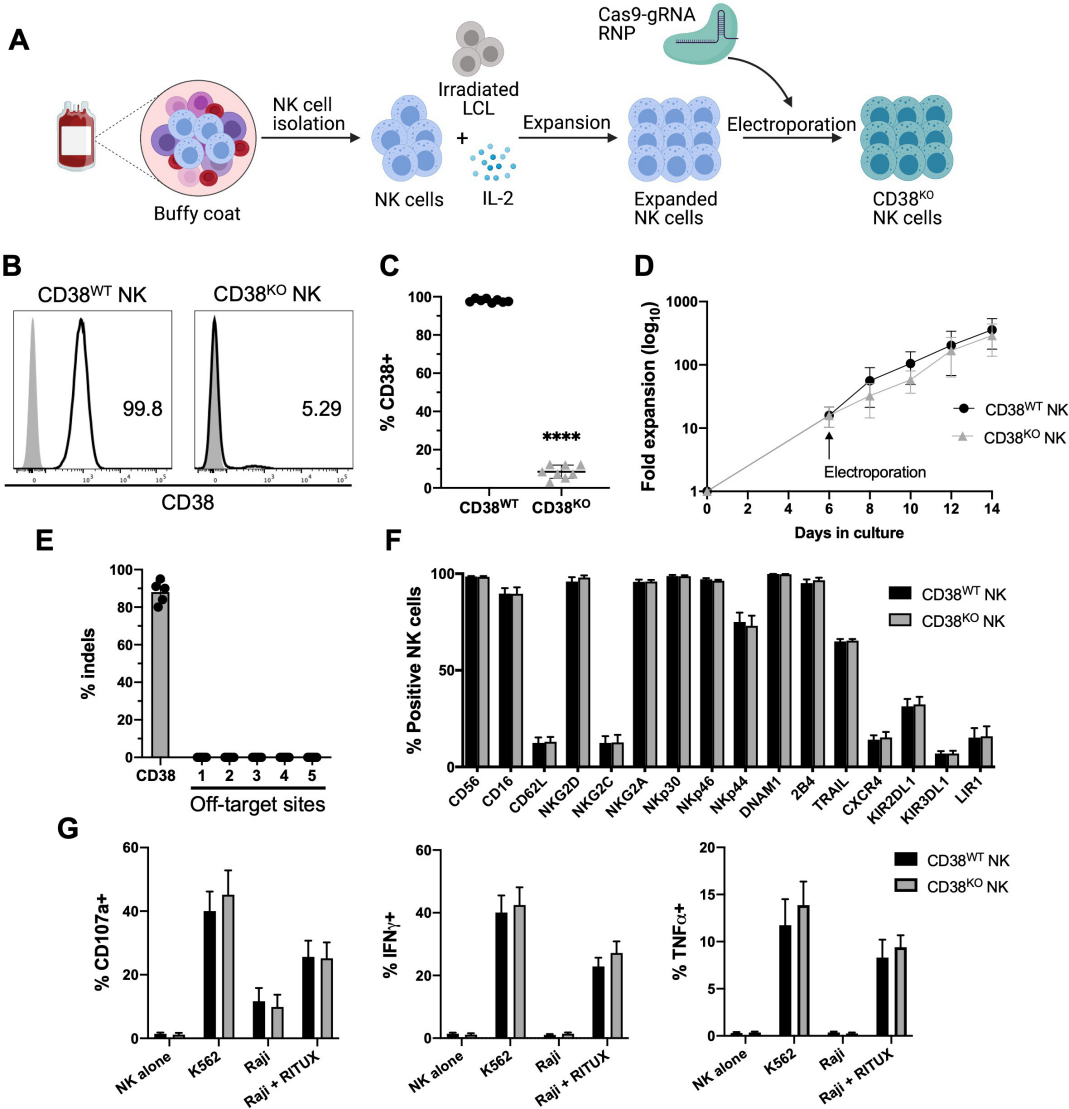
Highly efficient method for CD38 KO in ex vivo expanded NK cells. (A) Ex vivo expansion and CRISPR/Cas9 gene editing protocol. NK cells were isolated from healthy donor PBMCs and cocultured with irradiated LCL feeder cells and IL-2 for 1 week. Expanded NK cells were electroporated using precomplexed Cas9 and CD38-targeting gRNA. Edited NK cells were then expanded further in IL-2 containing media and analyzed for KO efficiency, phenotype, and function 7 days after CRISPR/Cas9 editing. CD38^WT^ NK cells were unmanipulated. (B) Representative histograms of relative expression of CD38 in CD38^WT^ and CD38^KO^ NK cells from one NK cell donor. (C) Pooled data showing relative expression of CD38 in CD38^WT^ and CD38^KO^ NK cells (n=8 donors). (D) Ex vivo fold expansion of CD38^KO^ NK cells compared with unedited CD38^WT^ NK cells (n=8 donors). (E) Assessment of off-target activity of the CD38-targeting gRNA/Cas9 RNP, shown are percentage of indels detected in CD38^KO^ NK cells at the CD38 on-target site as well as five in-silico-predicted off-target sites, as analyzed using the on-line ICE tool (n=5 donors). (F) Expression of a panel of NK cell surface markers examined by flow cytometry in CD38^KO^ and CD38^WT^ NK cells (n=6 donors). (G) NK cell degranulation (measured by CD107a expression), and intracellular IFN-γ and TNF-α expression examined by flow cytometry of CD38^KO^ NK cells compared with CD38^WT^ NK cells following coculture with K562 cells and Raji cells with and without rituximab (RITUX) (n=6 donors). Statistics determined with the Student’s t-test, two tailed, ****p<0.0001. Differences between CD38^WT^ and CD38^KO^ NK cells in panels F and G were not significant. ICE, inference of CRISPR Edits; KO, knockout; WT, wild type; NK, natural killer; PBMC, peripheral blood mononuclear cell; LCL, lymphoblastoid cell line.

CD38 is highly expressed in primary NK cells and was found to increase during ex vivo expansion (online supplemental figure 1). To protect NK cells from CD38 targeting, we optimized a non-viral Cas9/RNP system to disrupt expression of CD38 (figure 1A). Using this method, the mean CD38 KO efficiency in ex vivo expanded NK cells was approximately 92% (figure 1B, C). CD38^KO^ and unmanipulated CD38^WT^ NK cells exhibited similar viability (online supplemental figure S2) and capacity to expand to high numbers ex vivo (figure 1D). In CD38^KO^ NK cells, surface CD38 was reduced to a minimal level by 96 hours postediting, resulting in a stable CD38^KO^ phenotype that persisted for the remainder of the culture (online supplemental figure S2).

**Figure 2:**
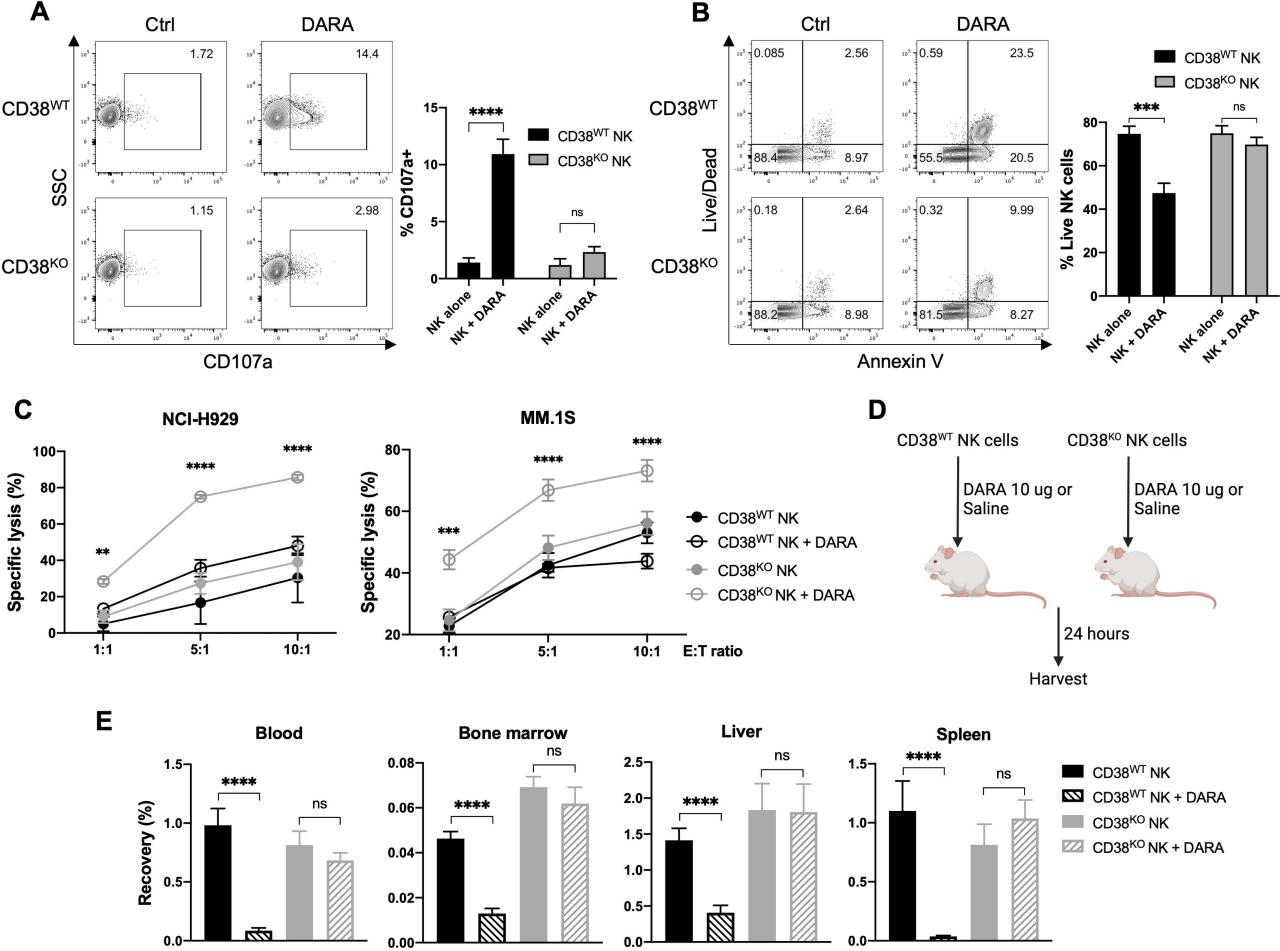
CD38 KO confers resistance to DARA-induced NK cell fratricide, and augments NK cell ADCC and in vivo persistence in the presence of DARA. NK cells were treated for 4 hours with 10 ug/mL of DARA, then examined by flow cytometry for (A) degranulation (measured by CD107a expression) and (B) viability (measured by Annexin V and Live/Dead staining) (n=6 donors). (C) ADCC mediated by CD38^KO^ and CD38^WT^ NK cells was assessed against NCI-H929 and MM.1S myeloma target cells at various effector-to-target (E:T) ratios (n=6 donors). (D) Schematic illustrating the experimental strategy to assess in vivo persistence of NK cells in the presence of DARA. (E) Recovery of human NK cells from blood, bone marrow, liver, and spleen of NSG mice in the presence or absence of DARA 24 hours after NK cell infusion (n=12 mice/group). Statistics determined with the Student’s t-test, two tailed, ns=not significant, **p<0.01, ***p<0.001, ****p<0.0001. ADCC, antibody-dependent cellular cytotoxicity; DARA, daratumumab; MM, multiple myeloma; KO, knockout; WT, wild type; NK, natural killer.

An analysis of in-silico-predicted off-target sites found no evidence of off-target activity from our CD38-targeting gRNA (figure 1E). Additionally, we evaluated potential phenotypic and functional consequences of CRISPR gene editing that could disrupt NK cell activation and target recognition. Flow cytometric analysis revealed CRISPR editing did not significantly alter the expression of a panel of NK cell surface receptors, including CD16 (figure 1F). We also conducted assays to assess the functional capacity of CD38^KO^ NK cells. Importantly, NK cell cytotoxicity, as measured by degranulation against K562 cells, and ADCC, as measured against rituximab-treated Raji cells remained high and was not significantly different among CD38^WT^ and CD38^KO^ NK cells (figure 1G). Production of IFN-γ and TNF-α, measured under the same conditions, was also similar in CD38^WT^ and CD38^KO^ NK cells (figure 1G). Taken together, this optimized Cas9/RNP platform permanently disrupts CD38 expression while preserving NK cell proliferative and functional capacity.

### CD38 KO protects NK cells from CD38 targeting and enhances DARA-mediated ADCC

DARA has been shown to induce NK cell fratricide in a CD16-dependent manner via ADCC directed at CD38-expressing NK cells.^23^ Dara-induced NK cell death can be abrogated by blocking DARA’s recognition of CD38 on the NK cell surface using DARA F(ab’)_2_ fragments.^23^ However, the clinical applicability of this approach appears limited, as F(ab’)_2_ mediated CD38 blockade occurs only transiently, preventing sustained in vivo protection from DARA-induced NK cell death (unpublished observations). To assess if permanent disruption of CD38 expression using CRISPR could protect NK cells from CD38 targeting, we evaluated degranulation and viability of CD38^KO^ and CD38^WT^ NK cells in the presence of DARA. The addition of DARA to NK cells led to increased degranulation by CD38^WT^ NK cells, as measured by CD107a expression, but did not significantly increase degranulation by CD38^KO^ NK cells (figure 2A). In line with these results, increased NK cell death and apoptosis were seen in DARA-treated CD38^WT^ NK cells, but not in CD38^KO^ NK cells (figure 2B).

Given in vitro data showing CD38 deletion protected NK cells from DARA-induced fratricide, we hypothesized that both NK cell persistence and cytotoxicity against MM target cells would be augmented in tumor-bearing mice receiving treatment with CD38^KO^ NK cells and DARA. To gage the impact of CD38 deletion on DARA-driven ADCC against MM target cells, we conducted in vitro NK cell killing assays against CD38-expressing MM cell lines with and without DARA. No significant difference was observed in the ability of CD38^WT^ and CD38^KO^ NK cells to lyse MM cells in the absence of DARA (figure 2C). In contrast, CD38^KO^ NK cells displayed significantly enhanced cytotoxicity compared with CD38^WT^ NK cells in the presence of DARA (figure 2C).

To determine whether CD38 deletion would also confer protection from DARA in vivo, we assessed the ability of adoptively infused human NK cells to persist in DARA-treated immunodeficient mice (figure 2D). DARA treatment led to a substantial reduction in the number of CD38^WT^ NK cells recovered from peripheral blood, bone marrow, spleen, and liver (figure 2E). In contrast, DARA treatment did not significantly decrease the number of CD38^KO^ NK cells recovered in any of the organs evaluated (figure 2E). Taken together, CD38 deletion in NK cells augments DARA-mediated ADCC of MM target cells in vitro by overcoming DARA-mediated fratricide, which enables NK cells to persist at significantly higher levels in vivo in DARA-treated mice.

### CD38^KO^ NK cells exhibit enhanced DARA-mediated anti-CD38 activity in vivo

**Figure 3:**
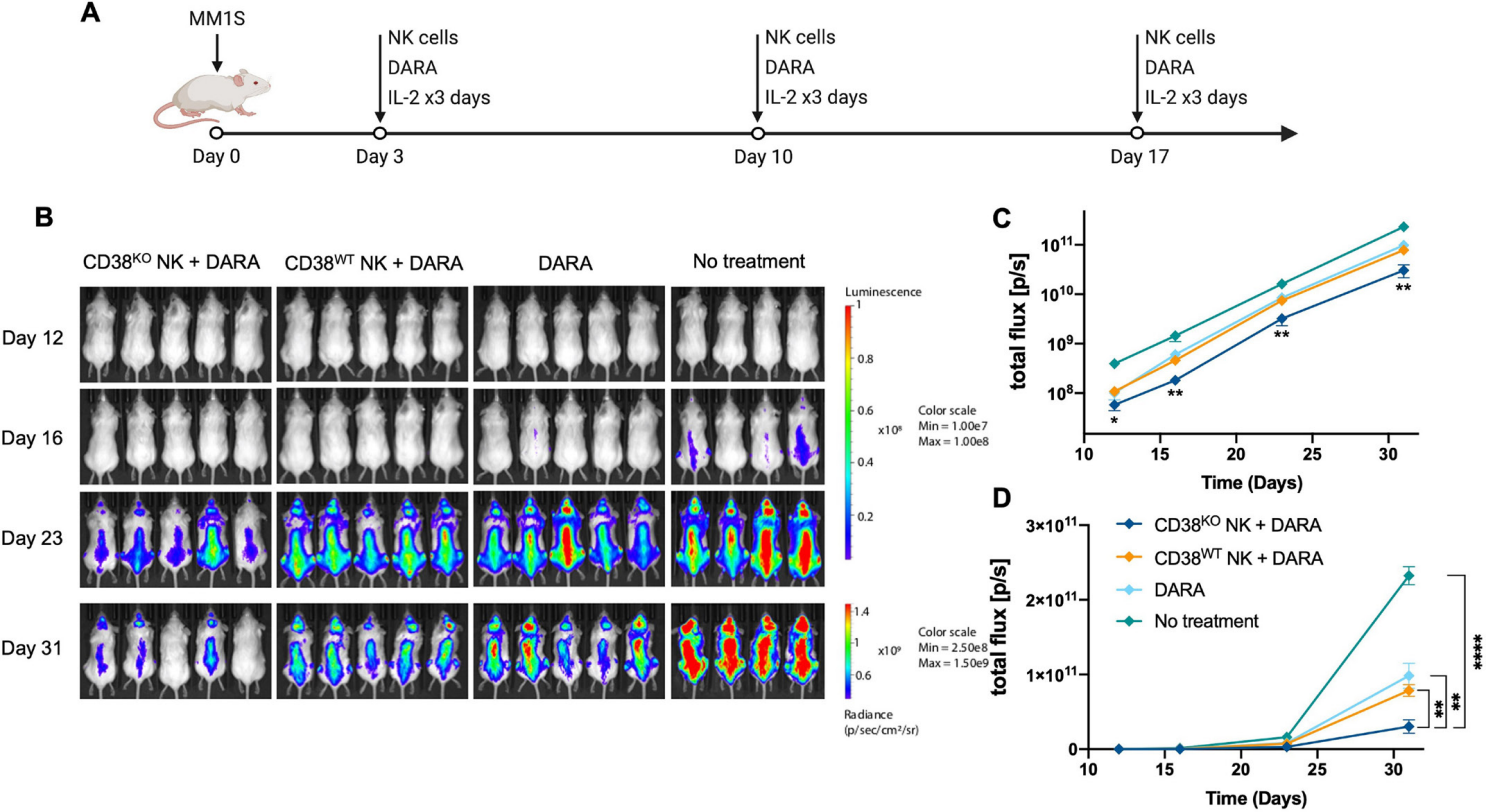
CD38^KO^ NK cells demonstrate enhanced anti-myeloma function in vivo. (A) Experimental schematic illustrating the treatment schedule. (B) Bioluminescence images show mice in each treatment group at 12, 16, 23, and 31 days after myeloma inoculation. (C) Logarithmic scale and (D) linear scale of bioluminescent quantification of MM.1S myeloma tumor growth across all time points (n=5 in all groups except n=4 in the no treatment group). Statistics determined with the Student’s t-test, two tailed, *p<0.05, **p<0.01, ****p<0.0001. DARA, daratumumab; KO, knockout; WT, wild type; MM, multiple myeloma; NK, natural killer.

To determine whether the enhanced ADCC capabilities of CD38^KO^ NK cells with DARA would translate to superior antitumor activity against myeloma cells in vivo, we tested the capacity of CD38^WT^ and CD38^KO^ NK cells to augment the antitumor activity of DARA in a MM.1S xenograft mouse model. Informed by a previously described model,^24^ we administered doses of DARA alone or in combination with either CD38^WT^ or CD38^KO^ NK cells adoptively infused at weekly intervals and monitored tumor growth by bioluminescence imaging (figure 3A). Administration of CD38^KO^ NK cells combined with DARA led to a greater reduction in MM.1S tumor burden compared with the control group and all other treatment groups (figure 3B–D). Thus, treatment with the combination of CD38^KO^ NK cells with DARA produced a significant additive antitumor effect that was more potent than DARA alone or CD38^WT^ NK cells combined with DARA. In summary, CD38^KO^ NK cells showed superior DARA-mediated antitumor activity against myeloma cells in both in vitro and in vivo compared with CD38^WT^ NK cells.

### Arming CD38^KO^ NK cells with the high-affinity CD16-158V receptor augments DARA-mediated ADCC

**Figure 4:**
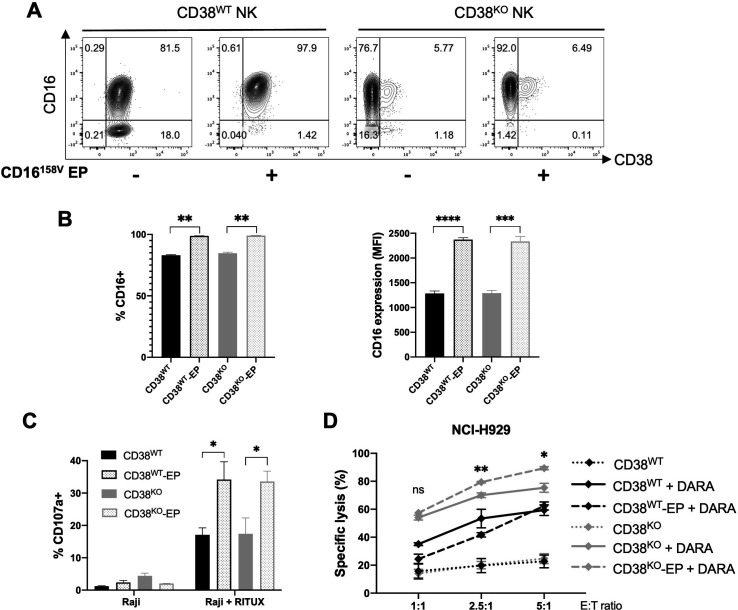
Functional capacity of CD38^KO^ NK cells can be altered by electroporation with CD16-158V mRNA. (A) Representative flow cytometry plots and (B), pooled data of relative expression of CD16 in CD38^WT^ and CD38^KO^ NK cells 24 hours after mRNA electroporation (n=3 donors). (C) NK cell degranulation (measured by CD107a) by CD16-158V mRNA-EP NK cells compared with non-EP NK cells following coculture with Raji cells with or without rituximab (RITUX) 24 hours after electroporation (n=3 donors). (D) DARA-mediated ADCC by CD16-158V mRNA-EP NK cells compared with non-EP NK cells assessed against NCI-H929 myeloma target cells at various effector-to-target (E:T) ratios 24 hours after electroporation (n=3 donors). Statistics determined with the Student’s t-test, two tailed, ns=not significant, *p<0.05, **p<0.01, ***p<0.001, ****p<0.0001. ADCC, antibody-dependent cellular cytotoxicity; DARA, daratumumab; EP; electroporated; KO, knockout; WT, wild type; NK, natural killer.

A naturally occurring variant of the CD16 antibody-binding receptor, that results from a phenylalanine (F) to valine (V) substitution at amino acid residue 158, has been shown to bind IgG with higher affinity and to induce more potent ADCC.^31^ We previously used mRNA electroporation to improve NK cell ADCC against rituximab-treated CD20^+^ lymphoma cells by delivering mRNA encoding the high-affinity CD16-158V receptor.^11^ To establish whether this transfection technique could also be used to enhance the ADCC capabilities of CD38^KO^ NK cells, we electroporated CD38^KO^ and control NK cells from low-affinity CD16-158F/F donors with CD16-158V mRNA and assessed CD16 expression and NK cell capacity to mediate ADCC. Electroporation resulted in a similar increase in CD16 expression in CD38^WT^ and CD38^KO^ NK cells (figure 4A, B). CD38^WT^ and CD38^KO^ NK cells that had been electroporated with CD16-158V mRNA exhibited a similar increase in degranulation against rituximab-treated CD20^+^ Raji cells (figure 4C). In contrast, in an assay using DARA-treated CD38^+^ NCI-H929 myeloma cells, CD38^KO^ NK cells transfected with CD16-158V mRNA displayed greater cytotoxicity than both CD16-158V-transfected CD38^WT^ NK cells and non-transfected CD38^KO^ NK cells (figure 4D). Taken altogether, these data establish that mRNA electroporation can be successfully utilized in CRISPR-edited NK cells to further modify their functional activity. Combining CD38 KO and CD16-158V mRNA transfection can be used to generate an NK cell population with augmented capacity for ADCC against CD38^+^ DARA-treated targets.

### A 2-in1 gene editing strategy for efficient gene KI targeted to the *CD38* locus

**Figure 5:**
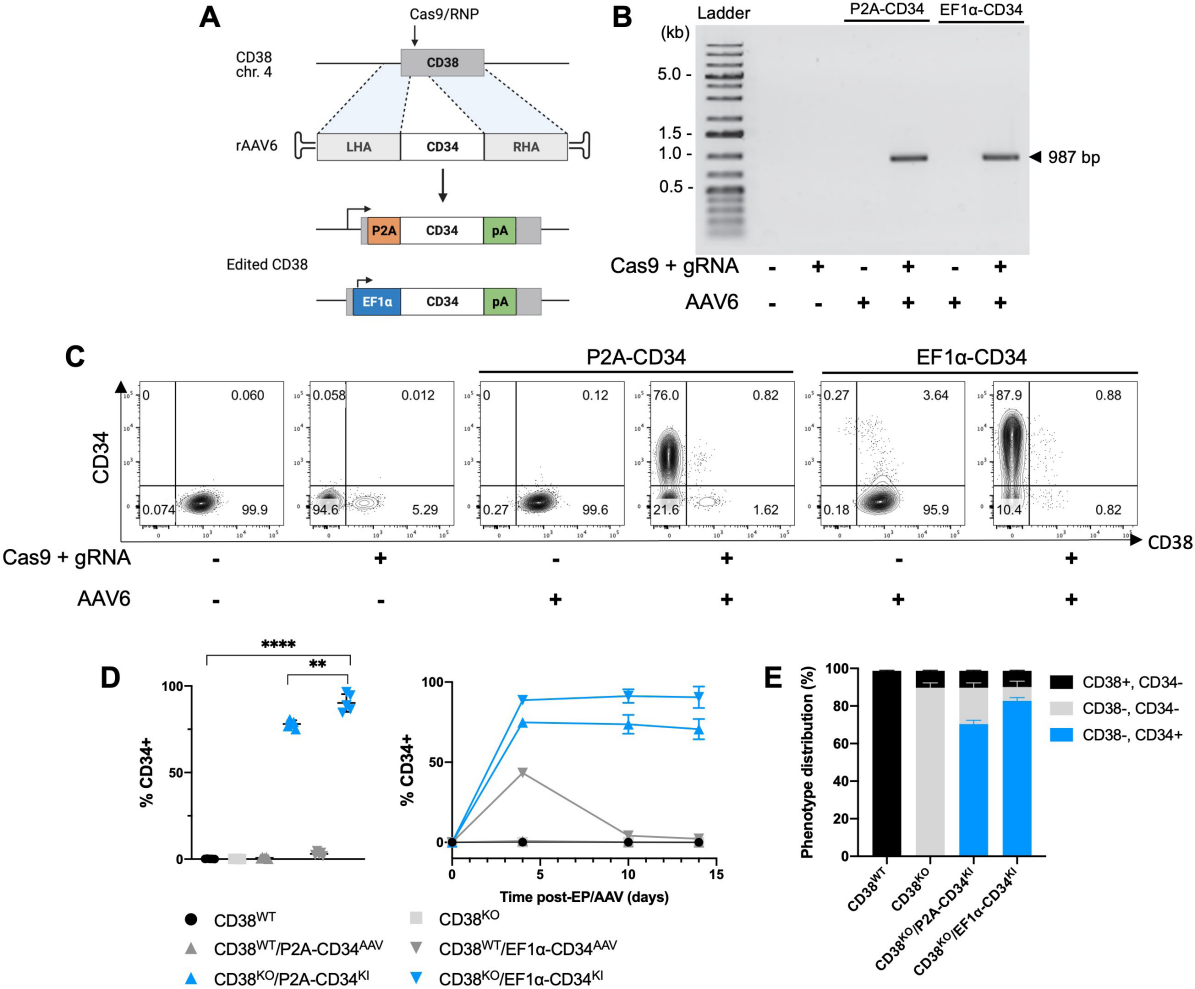
Gene knock-in (KI) targeted to the *CD38* locus results in highly efficient concurrent gene disruption and insertion. (A) Gene KI strategy to insert a truncated CD34 cassette into the C*D38* locus using Cas9/RNP and rAAV6. One of the CD34 cassettes contained an internal EF1α promoter and the other had a self-cleaving P2A peptide sequence in place of a promoter. (B) Representative gel image of PCR to detect site-specific integration of the CD34 transgene within the *CD38* locus. (C) Representative flow cytometry plots of CD38 and CD34 expression 7 days after gene KI targeted to the *CD38* locus. (D) CD34 expression assessed by percent positive and percent positive NK cells over time following Cas9/RNP electroporation and/or rAAV6 infection examined by flow cytometry (n=5 donors for CD38^KO^/CD34^KI^ groups and n=3 donors for all other groups). (E) Efficiency of 2-in-1 KO/KI determined by CD38 and CD34 expression in control and edited NK cells, examined by flow cytometry (n=3 donors). Statistics determined with the Student’s t-test, two tailed, **p<0.01, ****p<0.0001. KO, knockout; WT, wild type; NK, natural killer; LHA, left homology arm; RHA, right homology arm.

In addition to efficient CD38 gene disruption, we adapted our optimized platform to combine gene insertion with CD38 KO via HDR mechanisms. High efficiency gene KI within the *AAVS1* genomic safe harbor locus has been described in primary NK cells,^32^ but modifications made at safe harbor loci, by definition, are not expected to generate additional genomic or cellular effects beyond the gene KI.^33^ We sought to produce a simultaneous, 2-in-1 effect of gene KI coupled with CD38 KO by co-delivery of a CD38-targeting Cas9/RNP along with an rAAV6 encoding a DNA template for HDR containing a trans-gene of interest with flanking regions matching either side of the cleavage site in the *CD38* locus. We designed a CD34-encoding template containing an internal EF1α promoter as well as one containing a self-cleaving P2A peptide sequence in place of an internal promoter, designed to place CD34 under the transcriptional control of the disrupted *CD38* gene (figure 5A). Targeted integration of the CD34 transgene within the *CD38* locus was confirmed in CD38^KO^/CD34^KI^ NK cells by junction PCR (figure 5B). Combined CD38 KO/CD34 KI resulted in CD34 expression in approximately 85% and 75% of CD38^KO^ NK cells using the EF1α-containing and promoter-less HDR templates, respectively (figure 5C–E). A higher percentage and level of CD34 expression were seen with the EF1α-containing template. Similar to CD38 KO, CD34 KI was stable over time with both HDR templates (figure 5D). Transient CD34 expression was briefly observed in CD38^WT^ cells that were transduced with the EF1α promoter-containing AAV vector (figure 5D). In addition, no phenotypic alterations or impaired degranulation were observed in CD38^KO^/CD34^KI^ NK cells compared with unedited NK cells (online supplemental figure 3). Together, these data demonstrate a method to obtain high-efficiency generation of dual edited, KO/KI primary NK cells resulting in the delivery of a gene of interest to the KO’ed *CD38* locus.

### CD16-158V gene insertion coupled with CD38 KO boosts CD38-targeted ADCC

**Figure 6:**
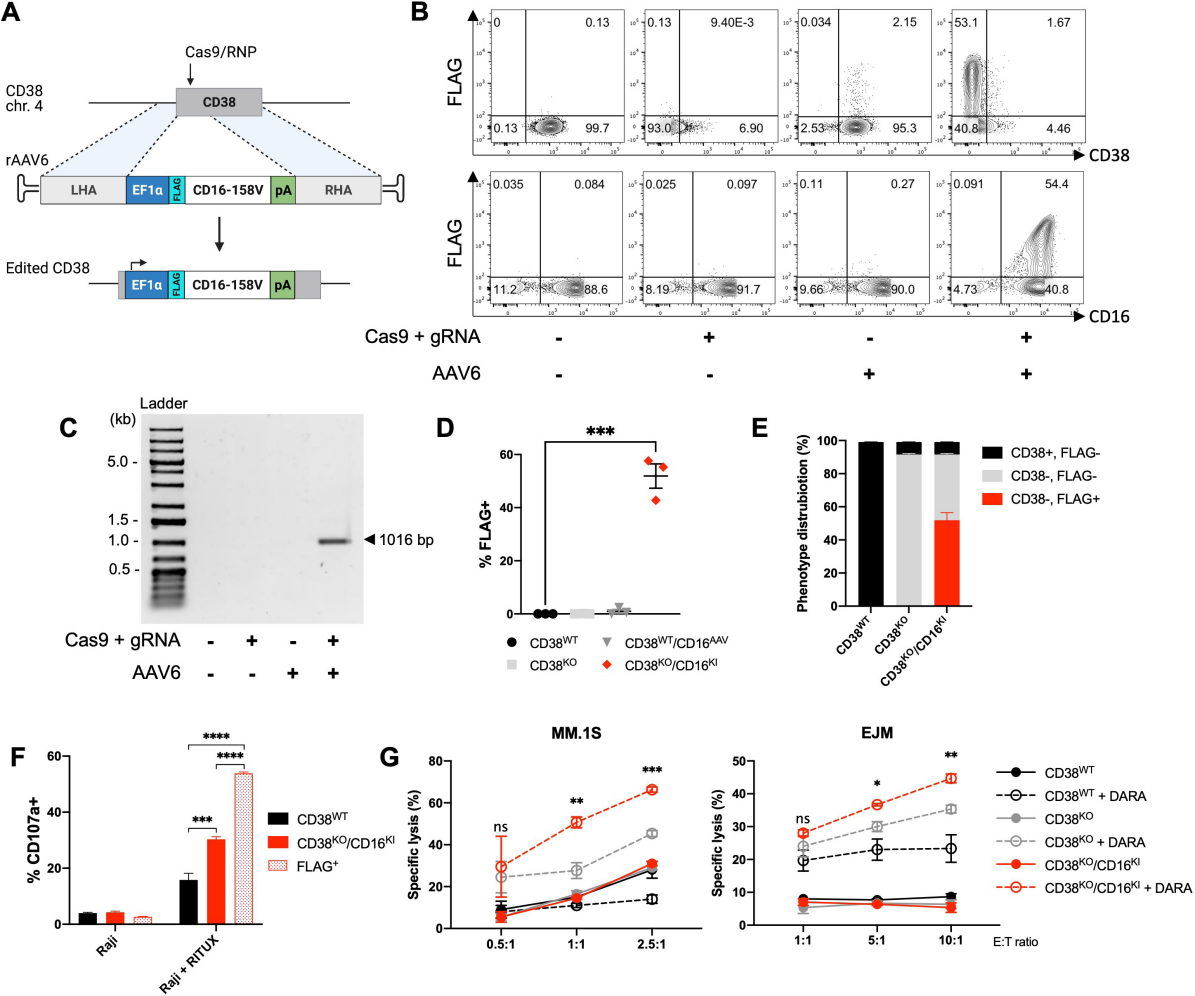
CD16-158V gene insertion coupled with CD38 knockout enhances CD38-targeted ADCC. (A) Gene knock-in (KI) strategy to insert a CD16-158V cassette containing a FLAG tag and EF1α promoter into the C*D38* locus using Cas9/RNP and rAAV6. (B) Representative flow cytometry plots of CD38, FLAG, and CD16 expression in NK cells 7 days after gene KI targeted to the *CD38* locus. (C) Representative gel electrophoresis image of PCR amplification products to detect site-specific integration of the CD16-158V transgene within the *CD38* locus. (D) FLAG expression assessed by percent positive NK cells 7 days following Cas9/RNP electroporation and/or rAAV6 infection examined by flow cytometry (n=4 donors). (E) Phenotype distribution of CD38 and FLAG in control and edited NK cells, examined by flow cytometry (n=4 donors). (F) NK cell degranulation (measured by CD107a) by CD38^KO^/CD16^KI^ NK cells compared with unedited control NK cells following coculture with Raji cells with or without rituximab (RITUX). CD107a was gated on bulk and FLAG-positive CD38^KO^/CD16^KI^ NK cells (n=3 donors). (G) DARA-mediated ADCC by CD38^KO^/CD16^KI^ NK cells compared with CD38^KO^ and control NK cells assessed against MM.1S and EJM multiple myeloma target cells at various effector-to-target (E:T) ratios (n=3 donors). Statistics determined with the Student’s t-test, two tailed, ns=not significant, *p<0.05, **p<0.01, ***p<0.001, ****p<0.0001. ADCC, antibody-dependent cellular cytotoxicity; DARA, daratumumab; KO, knockout; WT, wild type; MM, multiple myeloma; NK, natural killer; LHA, left homology arm; RHA, right homology arm.

To explore a functional application of our above-described 2-in-1 gene editing strategy, we sought to insert the high affinity CD16-158V receptor transgene within the *CD38* locus. We designed a CD16-158V-encoding template containing an internal EF1α promoter and the sequence for an N-terminal FLAG tag (sequence: DYKDDDDK), to allow expression of the inserted CD16-158V receptor to be easily distinguished from endogenous CD16 expression (figure 6A). Combined CD38 KO/CD16-158V KI resulted in CD16-158V expression in approximately 57% of CD38^KO^ NK cells, as determined by surface FLAG expression (figure 6B, D and E), with in vitro expansion and cell yields following the use of this platform being robust (online supplemental figure 4). Targeted integration of the CD16-158V transgene within the *CD38* locus was confirmed in CD38^KO^/CD16^KI^ NK cells by junction PCR (figure 6C). CD16-158V transgene expression was lower than that observed with the CD34 reporter, which may be attributable to underestimation of CD16-158V expression as measured via the surrogate FLAG tag or potentially altered cell surface recycling of the N-terminal FLAG-CD16 fusion protein. Since a robust and functionally relevant level of CD16-158V expression was achieved, no further attempts to investigate or optimize CD16-158V KI were pursued.

Degranulation experiments showed ADCC against rituximab-treated Raji cells was augmented in CD38^KO^/CD16^KI^ NK cells compared with unedited NK cells (figure 6F). Gating on FLAG-positive vs bulk CD38^KO^/CD16^KI^ NK cells in this ADCC assay revealed an even higher level of degranulation among FLAG/CD16-158V-expressing NK cells. Additionaly, in an assay using DARA-treated CD38^+^ MM.1S and EJM myeloma cells, CD38^KO^/CD16^KI^ NK cells displayed greater cytotoxicity than both undedited CD38^WT^ NK cells and CD38^KO^ NK cells (figure 6G). These data highlight an efficient 2-in-1 combined KO/KI strategy that can be used to generate functionally enhanced ex vivo expanded NK cells that are optimized for CD38-directed ADCC against myeloma cells.

### CD16 KI combined with CD38 KO leads to superior antimyeloma activity of NK cells *in vivo*

**Figure 7:**
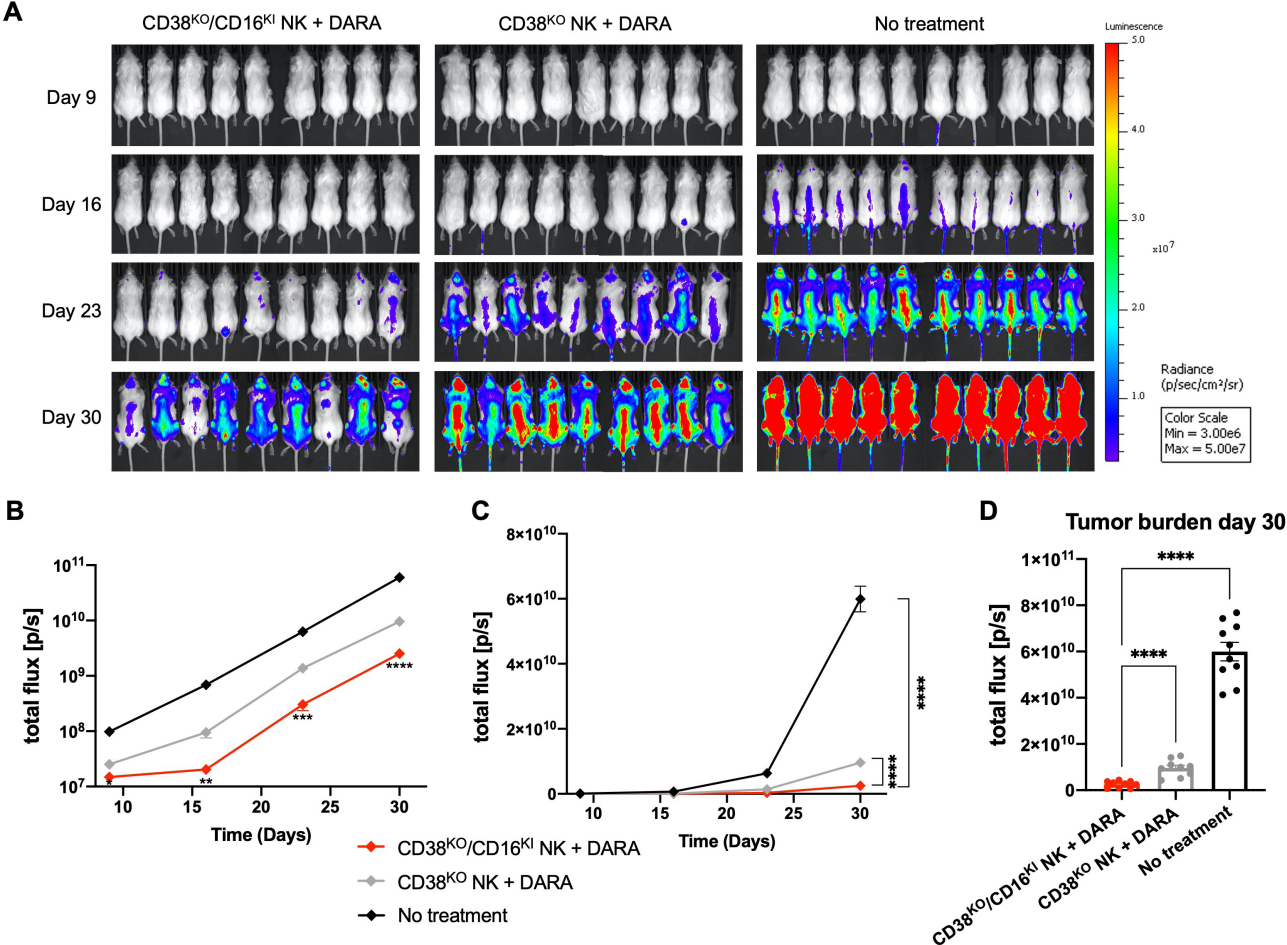
CD38^KO^/CD16^KI^ NK cells demonstrate enhanced anti-myeloma function in vivo. (A) Bioluminescence images show mice in each treatment group at 9, 16, 23, and 30 days after myeloma inoculation. (B) Logarithmic scale and (C) linear scale of bioluminescent quantification of MM.1S myeloma tumor growth across all time points (n=9 in treatment groups and n=10 in the no treatment group). (D) Bioluminescent quantification of MM.1S myeloma tumor growth shown in individual mice within each group at day 30. Statistics determined with the Student’s t-test, two tailed, *p<0.05, **p<0.01, ***p<0.001, ****p<0.0001. DARA, daratumumab; KO, knockout; KI, knock-in; NK, natural killer.

We next explored whether the superior in vitro DARA-mediated ADCC of CD38^KO^/CD16^KI^ NK cells would translate into an augemented antitumor effect in vivo. Using the same MM.1S xenograft mouse model presented above, the capacity of CD38^KO^/CD16^KI^ and CD38^KO^ NK cells to augment the antitumor activity of DARA was compared. Mice were administered doses of DARA in combination with adoptive infusions of NK cells at weekly intervals as illustrated in figure 3A. CD38^KO^/CD16^KI^ NK cells combined with DARA elicited a significantly greater reduction in MM.1S tumor burden compared with the CD38^KO^ NK cell treatment group (figure 7A–D), replicating in vivo the compelling data that was established in vitro. Adoptive infusions of CD38^KO^/CD16^KI^ NK cells combined with DARA, therefore, produced an additive antitumor effect beyond that seen in mice treated with DARA and CD38^KO^ NK cells. In summary, CD38^KO^/CD16^KI^ edited NK cells showed superior DARA-mediated antitumor activity against myeloma cells both in vitro and in vivo compared with CD38^KO^ NK cells, underscoring the clinical potential of adoptive immunotherapy strategies using this approach.

## Discussion

DARA is a CD38-targeting mAb with clinical efficacy in the treatment of MM, both as a single agent and as part of combination regimens.^20 21^ Among several mechanisms of action, DARA elicits efficient NK cell-mediated ADCC.^5^ High levels of CD38 expression in peripheral blood NK cells, however, predispose NK cells to DARA-induced depletion, leading to impaired ADCC against myeloma cells.^22 24^ Despite this limitation, the ADCC function of DARA can be rescued by minimizing CD38-targeting of NK cells by reducing or shielding NK cell surface CD38.^23 24^ Ex vivo modification to generate DARA-resistant NK cells for adoptive infusion with DARA is, therefore, an attractive strategy.

To enable the use of adoptively transferred NK cells to potentiate the activity of DARA, we sought to develop a CRISPR-based platform to genetically modify large numbers of ex vivo expanded human NK cells for clinical use. The platform we developed is clinically applicable and scalable, using electroporation of NK cells to deliver a CD38-targeted Cas9/RNP early during the ex vivo expansion process. This approach resulted in high level reduction of CD38 expression that was stable over time that did not compromise the proliferative or functional capabilities of the edited NK cells. CD38^KO^ NK cells showed reduced susceptibility to DARA-induced fratricide, improved in vivo persistence in the presence of DARA, and most importantly, enhanced DARA-mediated tumor control in vitro and in vivo in mice bearing MM.1S myeloma tumors.

These findings support previously reported data showing that NK cells with reduced CD38 following exposure to DARA^24^ or with CD38 shielded by F(ab’)_2_ fragments^23^ are protected from DARA-induced fratricide, enabling augmented DARA-mediated ADCC against tumor cells in vitro and in a preclinical animal model. However, in contrast to these approaches, disruption of CD38 expression using CRISPR is sustained over time, which potentially represents a more useful strategy that for translation into the clinic. Our findings corroborate a recent report describing the use of CRISPR to delete CD38 in NK cells, the results of which were recently published prior to this publication.^25^ Our optimized CRISPR platform utilized NK cells that were expanded using a GMP-compliant expansion protocol developed by our group.^10^ This protocol incorporates irradiated EBV-LCL feeder cells and IL-2 to expand large numbers of clinical grade NK cell ex vivo, which has been successfully used in the aforementioned NK cell clinical trial (NCT00720785). Furthermore, we delivered the Cas9/RNPs using an electroporation system that is scalable and can be easily transitioned to a clinical GMP-compliant platform. Importantly, given the high KO efficiency of our approach, there exists no need to enrich for the CD38^KO^ NK cells that have enhanced DARA-mediated antitumor responses, thus avoiding an additional manufacturing step. Our CRISPR approach merged with our preexisting GMP compliant method to manufacture large numbers of ex vivo expanded NK cells lays the groundwork for clinical translation of this highly efficient gene editing platform.

We sought to further enhance the immunotherapeutic potential of CD38^KO^ NK cells by combining CD38 KO with other techniques previously shown by our group to enhance their ability to mediate ADCC. Based on our previous work, we introduced the high-affinity antibody-binding CD16-158V receptor into NK cells via mRNA transfection.^11^ Clinically, the CD16-158V polymorphism has been associated with higher response rates to the anti-CD20 mAb rituximab in lymphoma patients,^34^ and our preclinical studies have demonstrated that forced expression of CD16-158V in NK cells boosts their ability to mediate ADCC.^11^ This is an appealing approach to bolster ADCC given only a minority of patients are homozygous for the high-affinity CD16-158V variant.^35 36^ We established the feasibility of transfecting CD38^KO^ NK cells with CD16-158V and further demonstrated that these genetically modified NK cells had augmented degranulation against malignant B-cells in ADCC assays using rituximab. In a similar fashion, we observed CD38^KO^/CD16-158V-transfected NK cells had superior DARA-mediated myeloma killing compared with CD38^WT^/CD16-158V-transfected and CD38^KO^ NK cells from low-affinity CD16-158F/F homozygous donors. These results highlight the potential of combinatorial KO/KI gene modifying strategies to enhance the tumor killing capabilities of NK cells.

As noted in our previous work, mRNA transfection results in only transient expression of transgenes of interest in NK cells.^11 12^ Therefore, to enable stable, long-lasting expression of a desired gene, we sought to develop a simplified strategy that would incorporate site-specific gene insertion into our CD38 KO platform. Recent advances in genome editing have enabled targeted gene delivery to the *AAVS1* safe harbor locus in primary NK cells^32^ and to strategic non-safe harbor loci in T cells, such as the T-cell receptor α constant (*TRAC*) locus.^37^ Efficient creation of a double strand break using Cas9 and target-specific guide RNA, as achieved with our CD38 KO method, generates a potential insertion site within the CRISPR disrupted CD38 domain for targeted gene delivery. Using rAAV6 to deliver a repair template containing a gene of interest flanked by segments that are homologous to the *CD38* locus, we successfully targeted a truncated CD34 reporter gene to *CD38*. This strategy resulted in stable CD34 expression in approximately 85% of CD38^KO^ NK cells using a repair template containing an EF1α promoter. Remarkably, a template lacking an internal promoter, designed to place the CD34 transgene under the transcriptional control of the CD38 promoter and regulatory sequences, also yielded a high level of stable gene expression, although slightly less than was observed with the EF1α-containing vector.

This efficient transgene delivery, reported here for the first time at the *CD38* locus, resulted in expression levels comparable to that reported in NK cells at the *AAVS1* locus,^32^ and to the best of our knowledge, is the first use of this approach in primary NK cells. This combinatorial genetic editing approach has advantages over either single gene KO or KI targeted to a safe harbor locus, as it has the potential to amplify the impact of the gene modification step on NK cell antitumor function beyond either approach alone. To move beyond proof-of-concept demonstrated using a control CD34 reporter, we provide evidence establishing this strategy can be used to insert functionally relevant cargo within the disrupted *CD38* locus. Insertion of CD16-158V augmented the ADCC function of CD38^KO^ NK cells against B-cell and myeloma tumor targets and elicited superior CD38-directed antitumor activity in vivo in a myeloma mouse model, underscoring the potential of this approach to generate modified NK cells that optimize CD38-directed immunotherapy. This streamlined platform also paves the way for combining CD38 KO with the KI of other strategic cargo such as receptors supporting NK cell homing, in vivo proliferation,^14^ and CD38-targeting chimeric antigen receptors (CARs). Furthermore, these results highlight the potential for an analogous approach coupling gene KI with KO of various other NK cell checkpoints and underscore the vast potential of modern gene editing techniques to aid in the development of a next generation of NK cells with improved antitumor efficacy.

In summary, we established an efficient gene editing platform designed to generate NK cells for use with the CD38-targeting mAb DARA. We showed that CD38 KO confers resistance to CD38-specific NK cell depletion and enhances DARA’s antitumor activity through augmented ADCC. We demonstrated that this platform can be readily combined with a clinically utilized ex vivo expansion technique to generate engineered NK cells for adoptive cell therapy applications. In addition, we extended these findings by developing an efficient 2-in-1 combined KO/KI strategy that generates CD38^KO^/CD16^KI^ NK cells that augment CD38-directed antitumor activity of primary human NK cells in vivo. The genetic modification techniques presented here form the basis for a streamlined strategy to explore this immunotherapy strategy in the clinic.

## Data Availability

All data relevant to the study are included in the article or uploaded as online supplemental information.
