# Supplementary material for: High-affinity CD16 integration into a CRISPR/Cas9-edited CD38 locus augments CD38-directed antitumor activity of primary human natural killer cells
Source: J Immunother Cancer. 2022 Feb 8;10(2):e003804. doi: 10.1136/jitc-2021-003804 (PMC8830298; doi:10.1136/jitc-2021-003804)
Supplement: Supplementary data [file jitc-2021-003804supp001.pdf]

Supplemental Figures and Tables

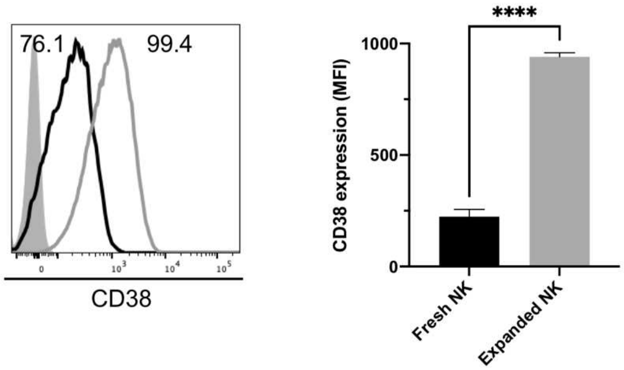

Supplemental Figure 1. CD38 is expressed by peripheral blood NK cells and increases with *ex vivo* expansion. Representative flow cytometry histograms and pooled data showing relative CD38 expression in freshly isolated NK cells (black) compared to NK cells after 7 days of *ex vivo* expansion with irradiated LCL feeder cells in IL-2 containing media (grey) (n = 3 donors). Statistics determined with the Student's t-test, two tailed, **** $p < 0.0001$.

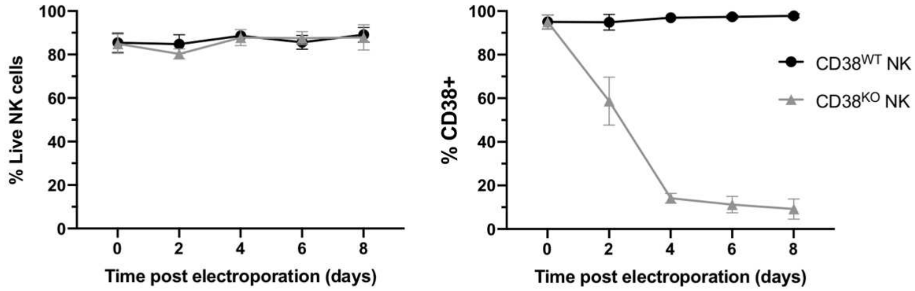

Supplemental Figure 2. NK cell viability and kinetics of CD38 expression following CD38 KO. Viability (determined by Live/Dead and Annexin V) and CD38 expression of *ex vivo* expanded $CD38^{WT}$ and $CD38^{KO}$ NK cells were examined by flow cytometry over time following CD38 KO (n = 6 donors).

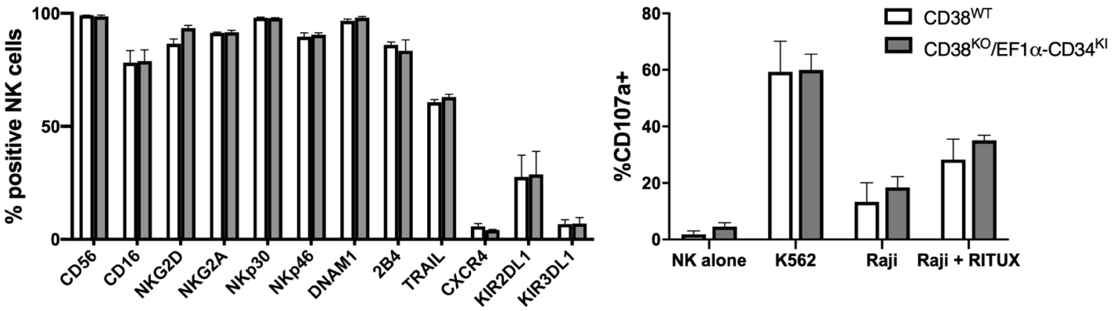

Supplemental Figure 3. Phenotypic and functional characterization of ex vivo expanded NK cells following combined CD38 KO/CD34 KI. Expression of a panel of NK cell surface markers examined by flow cytometry in CD38^{KO}/CD34^{KI} and unedited control NK cells (n = 3 donors). NK cell degranulation (measured by CD107a expression) was also examined by flow cytometry following coculture of NK cells with K562 and Raji tumor cells with and without rituximab (RITUX) (n = 3 donors).

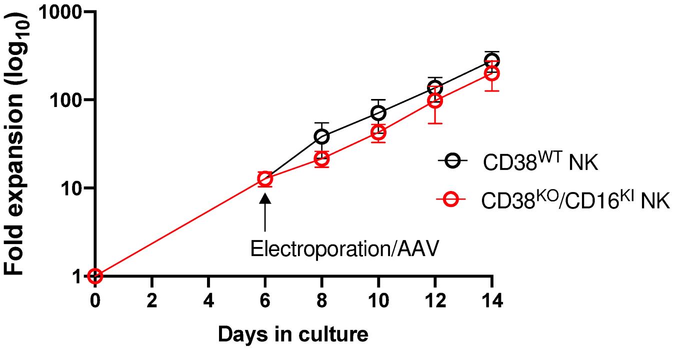

Supplemental Figure 4. Cell numbers during expansion of CD38^{KO}/CD16^{KI} and CD38^{WT} NK cells were tracked over time (n = 3 donors).

Primer name	Forward	Reverse
CD38	5’-ACCCCTGGTAGACTGCATGTT-3’	5’-TCTCTCTTAGCTCCCTTCTCCA-3’
OFF-1	5’-CTGGACTCAAGGGATGAGGACA-3’	5’-CTCGGAGAAGTTGGTGTTCCG-3’
OFF-2	5’-GTTTCCCTAAGCTTTGCTGGC-3’	5’-AAGGCCAGGAGGTTCCTTCA-3’
OFF-3	5’-CTTGACCTGTCAGCTTGGCTA-3’	5’-GAACCACTGGAACTCTTGGCA-3’
OFF-4	5’-GTGGGACAAGGGTCTTCACAG-3’	5’-GTGACCAGTATCATGCCACCT-3’
OFF-5	5’-TCTGGGAGGAAATTTGAGGGC-3’	5’-GCATTTCCCGCAGAGGAGA-3’

Supplemental Table 1. Primer sequences used for off-target analysis, including primer sets for the on-target CD38 site and 5 off-target sites.
